# 

**Published:** 1892-08-13

**Authors:** 


No. 307, Vol. XII.—August 13th, 1892.

[Registered at the General Post Office as a Newspaper for transmission in the United Kingdom and Abroad.]

WITH EXTRA NURSING SUPPLEMENT

Per Annum, 8s. 6d., post free.

Monthly Parts, 6d.; post free, 8d.

Ditto per Annum, 8s., post free.

### THE HOSPITAL

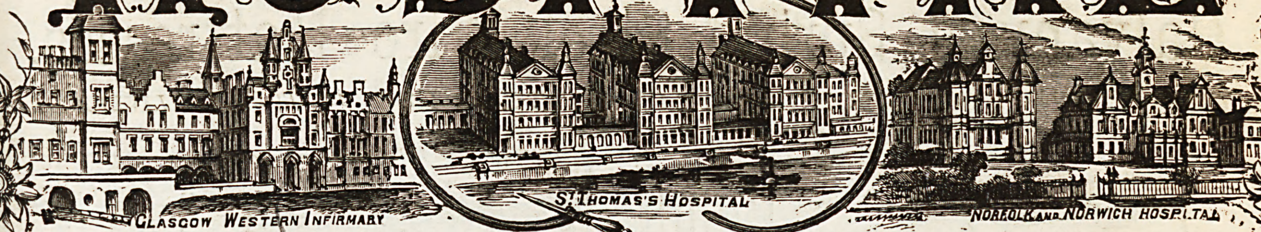

WITH EXTRA NURSING SUPPLEMENT.

No. 307. Vol. XII.] SATURDAY, AUGUST 13th, 1892. [TWOPENCE.

